# Food Safety Assessment of Commercial Genetically Modified Soybeans in Rats

**DOI:** 10.3390/foods11040496

**Published:** 2022-02-09

**Authors:** Huan-Yu Lin, Jiunn-Wang Liao, Ruo-Shiuan Chen, Chia-Hua Chang, Hui-Wen Chang, Shih-Chieh Chang, Wen-Shen Chu, Chien-Ku Lin, Hsin-Tang Lin

**Affiliations:** 1 Bioresource Collection and Research Center, Food Industry Research and Development Institute, Hsinchu 30062, Taiwan; hyl@firdi.org.tw (H.-Y.L.); chw10@firdi.org.tw (H.-W.C.); cws269@gmail.com (W.-S.C.); 2 Graduate Institute of Veterinary Pathobiology, National Chung Hsing University, Taichung 40227, Taiwan; jwliao@dragon.nchu.edu.tw (J.-W.L.); syrupruo@gmail.com (R.-S.C.); 3 Graduate Institute of Food Safety, National Chung Hsing University, Taichung 40227, Taiwan; fashion30935@gmail.com; 4 Department of Veterinary Medicine, National Chung Hsing University, Taichung 40227, Taiwan; scchang@dragon.nchu.edu.tw; 5 Department of Food Science and Technology, Hung Kuang University, Taichung 43302, Taiwan; cklin@sunrise.hk.edu.tw; 6 Department of Food Science and Biotechnology, National Chung Hsing University, Taichung 40227, Taiwan

**Keywords:** commercial GM soybeans, food safety, 90-day study, rats

## Abstract

Although the safety of commercial genetically modified (GM) soybeans has been well evaluated and GM soybeans are legally sold under government management, some consumers still have concerns about their safety. The objective of this study was to evaluate the safety of commercial GM soybeans sold in markets as a food source. In the present study, two commercial GM (GM-1 and -2) soybeans and one non-GM soybean were randomly purchased and subjected to a whole food toxicity assessment. Rats (SD), male and female, were divided into six groups (10/sex/group). Two dosages of 1 g/kg/day and 5 g/kg/day of soybeans were selected for the low- and high-dose groups. Rats were administered the soybeans via daily oral fed for 90 days. The results indicate that the body weight, organ weight, biochemistry, hematology, and urology showed no biologically adverse effects. At necropsy, no significant differences between organ weights were noted between the non-GM- and GM soybeans-treated groups. Moreover, no gross or histopathological lesions were observed in the high-dosage (5 g/kg/day) fed groups of the non-GM and GM soybean fed rats. In conclusion, this food safety assessment revealed that commercial GM soybeans are substantially equivalent to non-GM soybeans in rats.

## 1. Introduction

With the development of biotechnology in recent decades, research on genetically modified (GM) organisms has become more advanced. With a decrease in cultivated land and increasing populations unevenly distributed around the world, increasing crop yields through the use of GM plants has become an attractive option for handling these challenges. Today, soybean cultivated land measures up to 51% of the global area of GM crops. Moreover, in America and Brazil, the area used for sowing transgenic soybeans is greater than that used for traditional soybeans [1]. Soybeans are most commonly used for animal feed and pressing oil. Worldwide, 98% of soybeans are used for animal feed and 2% are manufactured into high-protein bean products such as isolated soy protein, concentrated soy protein and soy flour [2]. In Asia, approximately 10% of soybeans are used for food manufacturing, such as bean curd (tofu), soy milk, and other processed soybean products. In Taiwan, on the other hand, soybeans are consumed for food at about 30.58 g/person/day (0.51 g/kg body weight/day) [3], which is higher than in western countries (less than 1 g soybean/person/day) [4], and most soy is imported.

According to the Food and Agriculture Organization (FAO) [5] and the Organization for Economic Co-operation and Development (OECD) [6], GM food is determined to be substantially equivalent to traditional food when evaluating the food’s characteristics, semblance, agricultural properties, component analysis, nutrition analysis, and allergens. As stated in the codex guidelines, The European Food Safety Authority (EFSA) has proposed the inclusion of key allergens in comparative analysis, especially allergenic crops such as soybeans [7,8]. The rationale is that changes in the levels of these proteins may lead to altered allergenicity in transgenic plants, an unintended effect of genetic modification [9,10]. Soy allergy primarily affects young children, most of whom eventually outgrow allergy symptoms [11]. Due to known allergenicity, researchers have focused on protein in soybean seed (*Glycine max*) [11,12] and the existence of many GM varieties [13]. To date, eight allergenic soy proteins have been identified [14,15]. However, based on our knowledge, it is still difficult to obtain recognized conclusions regarding the allergenicity of GM foods by comparing endogenous allergen levels with those in conventional foods [16]. The allergenicity is compared between transgenic and conventional soybeans with respect to the foci Gly m 4 and Gly m Bd 30K in human serum. Patients allergic to soy and those with a history of hypersensitivity to birch pollen and bovine casein might not experience further increased allergic reactions after exposure to GM soybeans [17].

Nevertheless, GM food products are existing controversy among policymakers, scientists and consumers including agricultural, environmental, ecological and health risks [18]. GM organisms and their derived food and feed products must undergo risk assessment and regulatory approval before they can be sold on the market [19]. Current approvals of GM soybeans in Taiwan consist of 17 in single events and 12 in stacked events. Most of them are herbicide-tolerant. For single events, there are 2,4-D, dicamba, glufosinate, imidazolinone, glyphosate, and isoxaflutole herbicide tolerances [20]. Although several researchers have confirmed the GM soybean to be substantially equivalent to traditional non-GM soybeans, the use of GM soybeans in food is still disputed in Europe and Taiwan [21,22]. Nowadays, in vivo experiments should be used to assess whether post-market GM food and feed affect animal health or not [23].

The objective of this study was to assess the food safety of commercial GM soybeans sold in markets. For this aim, a 90-day feeding study in rats is a widely accepted method for the safety evaluation of GM foods in Taiwan and is also requested by the EFSA [23]. Two commercial GM soybeans and one non-GM soybean were used to conduct the food safety assessment in rats for the 90-day feeding study. The evaluated items included DNA isolation and PCR assay of common GM genes in vitro and clinical observations consisting of body weight, food consumption, urinalysis, hematology, coagulation, serum chemistry, organ weight, and gross and histopathological examinations in rats.

In the global regulatory system, in order to carry out the toxicological trials before approval is obtained, seed companies only use the GM soybean seeds that have been developed but not yet hybridized. This means that such safety trials are not performed using commercially grown GM soybean. However, most soybeans purchased by consumers are mixed GM soybeans with different transgenic events (known as stacked traits), and the proportion and quantity of the original single event GM soybean cannot be ruled out. The safety assessment presented in this study is mainly from the consumer’s dietary scenario point of view, thus it takes into consideration the GM soybeans that consumers can directly purchase from the market.

## 2. Materials and Methods

### 2.1. Soybean Samples and Preparation

Non-GM soybeans (imported from DG Global Inc., Toronto, ON, Canada) and two GM soybeans (GM-1 and GM-2 samples, imported from the United States) were purchased from retailers in Taiwan. The soybeans were fully matured at the R8 stage before harvest and then export to Taiwan [17]. Soybean samples were steamed for 20 min at 121 °C by autoclave and then dried for 24–72 h in a dryer. In order to avoid crushing the oil out of the soybeans, soybean samples were stored in a refrigerator at −20 °C and then were ground to a fine powder using a grinding machine and passed through a 40-mesh screen.

### 2.2. DNA Isolation and PCR Assay

To confirm the transgenic genes of stacked GM soybeans, the samples were well blended and mixed in a high-speed blender (model 6640, Oster, Boca Raton, FL, USA). A total of 100 mg of soy powder of one non-GM soybean and two GM soybeans were collected for DNA isolation using the DNeasy plant mini kit (Qiagen Inc., Valencia, CA, USA).

The PCR primers were designed for the CaMV35S promoter (P35S), the CaMV35S terminator (T35S), the nos-terminator of *Agrobacterium tumefaciens* stain CP4 (T*nos*), the *cp4epsps* gene of *A. tumefaciens* stain CP4 (*cp4 epsps*), the *dmo* gene derived from *Stenotrophomonas maltophilia* strain DI-6 (*dmo*), the *cry1Ab/cry1Ac* gene derived from *Bacillus thuringiensis* subsp. Kurstaki strain HD73 (*cry1Ab/Ac*), the *pat* gene derived from *Streptomyces viridochromogenes*, the *gm-hra* gene derived from *Glycine max*, the *epsps* gene with double mutates from *Zea mays* (*2mepsps*), the *lectin* gene from *Glycine max,* the *crs1-2* gene derived from *Arabidopsis thaliana* (*gm-hra*), and the *Pj.D6D* gene derived from *Primula juliae* (*Pj.D6D*). Controls used in this study were (i) non-GM soybean (Ecocert Canada, 10145-14-01); (ii) certified reference soybean materials: A2704-12 (AOCS 0707-B6), CV127 (AOCS 0911-C), FG72 (AOCS 0610-A2), GTS 40-3-2 (ERM-BF410gk), MON87701 (AOCS 0809-A), MON87708 (AOCS 0311-A), MON87769 (AOCS 089-B) and 305423 (ERM-BF426d). The limit of detection for the test genes was 0.1%. The PCR conditions were as previously described [24].

### 2.3. Animals

Five-week-old SPF male and female Sprague Dawley rats were purchased from Biolasco Taiwan Co., Ltd. (I-Lan, Taiwan). The environmental conditions in the housing facility were maintained at 21 ± 2 °C and 50–70% humidity under a 12 h light/12 h dark cycle. Autoclaved Rat Chow (Purina 5010, St. Louis, MO, USA) and reverse-osmosis water were available ad libitum. This experimental plan was approved by the animal care and use committee of National Chung Hsing University (approval number: IACUC 104-021). A Guideline for the Care and Use of Laboratory Animals [25] was followed for animal feeding and housing procedures.

### 2.4. Experimental Design

The whole food 90-day feeding study for non-GM and GM soybeans was conducted in accordance with the experimental guidance of EFSA [23]. Non-GM and GM soybeans were diluted in autoclaved reverse-osmosis water. Rats were labeled by picric acid staining on the back and with individual cage cards. They were randomly assigned to a low-dose group (1 g/kg) or a high-dose group (5 g/kg) for GM or non-GM soybeans for 90 consecutive days feeding by daily gavage. There were 20 rats in each group, half male and half female, and their body weight did not exceed 20% of the average body weight of each sex at the beginning of the experiment.

### 2.5. Clinical Observations

Rats were observed daily for clinical signs before gavage. For feed consumption recording, rats were housed individually in cages with a feeding box. Body weight and feed consumption were measured weekly. At the end of feeding (day 91), all rats were fasted overnight and sacrificed in an inhalation chamber (MSS 003, Benchtop Small Animal Anesthesia Unit, Oxford, UK) under 2% isoflurane (Halocarbon Laboratories, River Edge, NJ, USA) anesthesia.

### 2.6. Hematology and Coagulation

Rat blood was collected from the abdominal aorta and put into anticoagulative tube (K3 EDTA syringes) (Vacutainer, Franklin Lakes, NJ, USA). The red blood cell (RBC) count, hematocrit (HCT), hemoglobin (HGB), mean corpuscular hemoglobin (MCH), mean corpuscular volume (MCV), mean corpuscular hemoglobin concentration (MCHC), and platelet (PLT) and white blood cell count (WBC) were evaluated with a hematology analyzer (Sysmex K-4500, Toa Medical Electronics, Kobe, Japan). Rat blood smears were stained by hematoxylin stain kit (A.J.P. Scientific, Clifton, NJ, USA) and the differential white blood cell counts (neutrophils, lymphocytes, monocytes, eosinophils and basophils) were observed by microscope (Olympus BX50 Microscope, Tokyo, Japan). Prothrombin time (PT), activated partial thromboplastin time (APTT), and fibrinogen (FBG) parameters were measured by using a coagulation analyzer (AMAX 200, Trinity Biotech, Bray, Ireland).

### 2.7. Serum Chemistry

Serum albumin, alkaline phosphatase (ALP), amylase, alanine aminotransferase (ALT), aspartate aminotransferase (AST), blood urea nitrogen (BUN), creatinine, creatine kinase (CK), glucose, lactate dehydrogenase (LDH), triglycerides (TG), total cholesterol (TC), total protein (TP), chloride ions (Cl^−^), calcium (Ca^2+^), potassium (K^+^), magnesium (Mg^2+^), sodium (Na^+^), and inorganic phosphorus (P^3−^) were detected using a clinical chemistry analyzer (Chiron Diagnostics Corporation, Oberlin, OH, USA).

### 2.8. Urinalysis

Urinalysis was assessed on day 0 and day 90 before sacrifice. Urine volume, specific gravity, bilirubin, pH, protein, glucose, ketones, nitrite, urobilinogen, and occult blood (Oc. blood) were tested by a Clinitex 100 Urine Chemistry Analyzer (Miles Inc. Diagnostic Division, Elkhart, IN, USA). Urinary sediments of RBC, leukocytes, epithelial cells (epithelia), casts, and crystals were observed by optical microscope (Olympus BX50F4, Tokyo, Japan).

### 2.9. Gross and Histopathological Examinations

Thorough necropsies were examined on all animals. Organs (brain, heart, thymus, liver, spleen, kidneys, adrenal glands), testes (males), and ovaries (females) were removed, weighed, and fixed in 10% buffered formalin. The organs of the control and the two high-dose (5 g/kg) GM soybean and one non-GM soybean groups, including the digestive system (esophagus, liver, stomach, duodenum, jejunum, ileum, cecum, colon, rectum—including Peyer’s patches, salivary glands, and pancreas), urinary system (urinary bladder and kidneys), respiratory system (larynx, lungs, trachea, and pharynx), cardiovascular system (aorta and heart), endocrine system (adrenal glands, pituitary gland, thyroid gland, and parathyroid glands), hematopoietic system (spleen, thymus, mandibular lymph node, mesenteric lymph node, and bone marrow), musculoskeletal system (skeletal muscle, femur/knee joint and sternum), nervous system (brain, (including cerebrum and cerebellum), spinal cord (cervical, thoracic, lumbar, and sciatic nerve), the male reproductive system (testis, epididymis, prostate and seminal vesicles) and female reproductive system (mammary glands, ovaries, uterus and vagina), skin and eyes (including retina and optic nerve) were trimmed according to the revised guidelines for organ sampling and trimming in rats and mice for histopathological examination [26]. For semi-quantitative grading, lesion severity was graded using the criteria developed by Shackelford [27]. Lesion severity was graded as follows: 1 = minimal (<1%); 2 = slight (1–25%); 3 = moderate (26–50%); 4 = moderate/severe (51–75%); and 5 = severe/high (76–100%). The lesion incidence was expressed as the number of affected rats out of the total number of rats examined.

### 2.10. Statistical Analysis

Data are presented as mean ± standard deviation (SD). Comparisons were designed to determine whether differences were attributable to non-GM and GM soybean-fed rat groups at the same dosage. Excel (Microsoft, Seattle, WA, USA) was used for statistical analysis, and statistical significance levels (*p* < 0.05) were determined by two-tailed tests and paired comparisons were made in the 1 g/kg and 5 g/kg groups.

## 3. Results

### 3.1. GM Soybean Confirmed by PCR for Transgenic Genes

**Figure 1:**
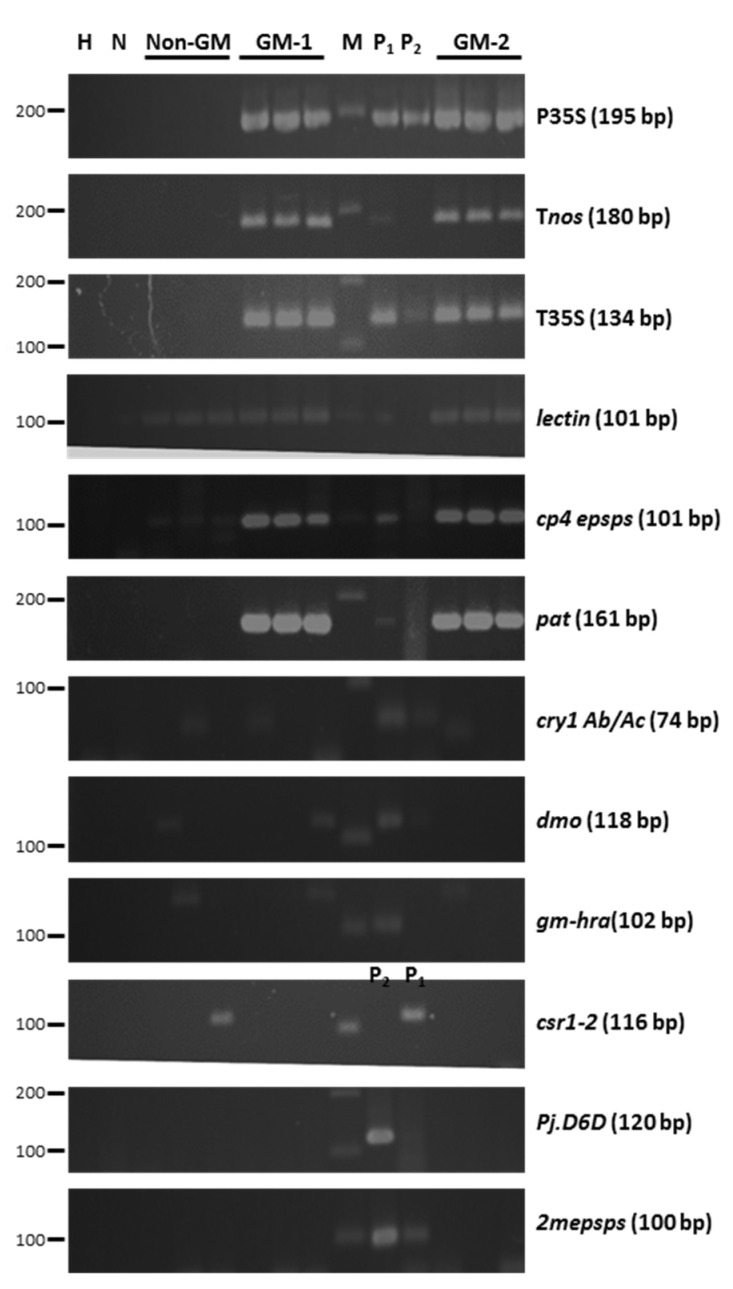
Screening of soybean products for foreign transgenes. DNA was isolated from the non-GM, GM-1, and GM-2 soybeans. The foreign transgenes were amplified using PCR for the P35S, the T*nos*, the T35S, the *lectin* gene, the *cp4 epsps* gene, the *pat* gene, the *cry1Ab/cry1Ac*, the *dmo* gene, the *gm-hra* gene, the *csr1-2* gene, the *Pj.D6D* gene, and the *2mepsps* gene. The detection limit was 0.1%. H is for ddH2O, N is for non-GM soybean, M is for DNA ladders, P_1_ is for 0.1% reference material of GM soybean, and P_2_ is for 0.01% reference material of GM soybean.

The soybean products were examined for transgenes by PCR assays. No transgenic gene, except the soybean-specific lectin gene, was detected in the non-GM soybeans. The P35S, T35S, T*nos*, *cp4 epsps*, *pat*, and soybean-specific *lectin* gene were detected in the GM soybean. The *cp4 epsps* gene, which produces the 5-enolpyruvoylshikimate-3-phosphate synthase (EPSPS) enzyme, is present in transgenic soybean events GTS 40-3-2 (MON-Ø4Ø32-6, Roundup Ready™), MON89788 (MON-89788-1, Genuity^®^ Roundup Ready 2 Yield™), and MON87705 (MON-877Ø5-6, Vistive Gold™) for glyphosate herbicide resistance. The *pat* gene, which produces the phosphinothricin acetyltransferase (PAT) enzyme, is present in transgenic soybean events A2704-12 (ACS-GMØØ5-3, Liberty Link™), A5547-127 (ACS-GMØØ6-4, Liberty Link™), DAS68416-4 (DAS-68416-4, Enlist™), SYHT0H2 (SYN-ØØØH2-5), DAS44406-6 (DAS-44406-6), and DAS81419 (DAS-81419-2) to eliminate the herbicidal activity of glufosinate (phosphinothricin) herbicides by acetylation (Figure 1).

### 3.2. Signs, Body Weight and Food Consumption

**Figure 2:**
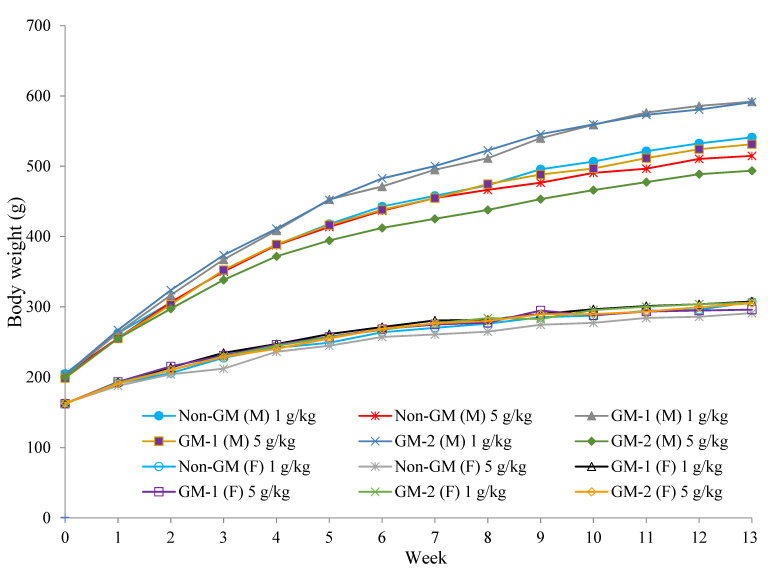
Body weight change in rats fed with non-GM and GM soybeans for 90 days. M: Male rats; F: Female rats. Data are expressed as the mean ± SD (n = 9–10).

No significant signs of toxicity were found in the male or female rats during the whole experimental period. However, two male rats, one in the GM-1 (1 g/kg) group and one in the GM-2 (5 g/kg) group, were found dead within one week because of gavage error. After 35 days, increased body weight and body weight gain in male rats were observed in the GM-1 (1 g/kg) and GM-2 (1 g/kg) groups when compared with the non-GM group. No difference in body weight was observed in female rats (Figure 2). Increased food consumption in male rats was observed in the GM-1 (1 g/kg) and GM-2 (1 g/kg) groups during some interval weeks when compared with the non-GM group. Increased feed efficacy was observed in male rats in the GM-1 (1 g/kg) group when compared with the non-GM group (Table 1).

### 3.3. Hematology and Blood Coagulation Examinations

In male rats, when compared with the non-GM group, a higher count of RBCs (10^6^/μL) was found in the GM-2 (5 g/kg) group. HGB was increased in the GM-1 (5 g/kg) and GM-2 (5 g/kg) groups, HCT was increased in the GM-1 (5 g/kg) and GM-2 (1, 5 g/kg) groups, blood coagulation was exhibited higher APTT in male rats in the GM-2 (1 g/kg) group, the WBC count (10^3^/μL) was decreased in the GM-1 (1 g/kg) and GM-2 (1 g/kg) groups, and eosinophils were decreased in the GM-2 (5 g/kg) group. In female rats, a higher count of RBCs (10^6^/μL) was found in the GM-1 (5 g/kg) group and monocytes and eosinophils were increased in the GM-2 (5 g/kg) group when compared with the non-GM group (Table 2 and Table 3).

### 3.4. Clinical Biochemistry and Urine Chemistry Examinations

In male rats, in comparison with the non-GM group, creatinine was increased in the GM-2 (1 g/kg) group and LDH and TG were increased in the GM-2 (5 g/kg) group (Table 4). In female rats, in comparison with the non-GM group, albumin was increased in the GM-1 (5 g/kg) and GM-2 (5 g/kg) groups, creatinine was increased in the GM-1 (5 g/kg) and GM-2 (5 g/kg) groups, glucose was decreased in the GM-2 (5 g/kg) group, and total cholesterol was increased in the GM-2 (5 g/kg) group. The Mg^2+^ level was increased in the GM-1 (5 g/kg) group, and P^3−^ was increased in the GM-2 (1 g/kg) group. However, these statistical differences were not dose-dependent, and these values were within the historical control values for normal age-matched (8–16 weeks of age) [28]. Therefore, these results were not considered biologically meaningful in male and female rats (Table 5). In urinalysis, GM and non-GM treated groups showed normal values and no significant differences (data not shown).

### 3.5. Organ Weight and Pathological Examinations

In male rats, adrenal gland weight was increased in the GM-1 and GM-2 (5 g/kg) groups when compared with the non-GM group. No other significant differences in organ weights were observed (Table 6). Furthermore, neither gross nor histopathological examination showed biologically significant lesions in the high-dose (5 g/kg) non-GM, GM-1, or GM-2 group. Some of the non-specific lesions that were observed during pathological examination included mononuclear cell infiltration of the Harderian gland, heart and prostate gland; cystic kidneys, islets fibrosis; serosal fibrosis in the spleen, and granulomas of the lungs that might have been caused by choking induced during gavage of the soybeans. These lesions were randomly observed among non-GM, GM-1 and GM-2 soybean-treated rats. Finally, in male or female rats, no apparent gross or microscopic lesions can be attributed to feed with non-GM, GM-1 or GM-2 soybean (Table 7).

## 4. Discussion

In this study, one non-GM soybean and two GM soybeans sold in markets were used for a 90-day feeding toxicity study. In Figure 1, the soybean products were further examined for transgenes by PCR assays. No transgenic genes except the soybean-specific *lectin* gene were detected in the non-GM soybeans. The P35S, T35S, T*nos*, *cp4 epsps*, *pat*, and soybean-specific lectin genes were detected in the GM soybeans, indicating that these soybeans contained glyphosate and glufosinate herbicide-resistant events.

Of the four biotech (GM) crops, including soybeans, maize, cotton, and canola, GM soybeans are the only ones that have increased in total hectares between 2014 and 2015. The reason for the decrease in maize, cotton, and canola is due to lower prices [1]. It has been shown that current approvals of GM soybeans in Taiwan amount to 16 in single events and that most of them are glyphosate and glufosinate herbicide-tolerant events [20].

GM crops still face challenges in food safety management, as some scientists insist that the unintended effects of GM crops due to the insertion of foreign genes may remain undetected [22,29]. Nowadays, many methods have been developed, validated and harmonized globally, with a focus on GMO detection [30,31]. Owing to the high rate of cellular proliferation and unique differentiation of mammalian testis, this sensitive organ can detect cellular and molecular changes when exposed to toxicants [32]. Thus, many scientists and researchers are concerned that GM foods may have negative effects on the male reproductive system. In addition, no significant differences in body weight, hematology, serum chemistry, or related side effects were found in the reproductive system of male rats fed with a GM corn diet [33].

The concept of substantial equivalence is proposed as the starting point for safety evaluations of GM crops. In recent years, international GMO and derived food safety assessments have been based on FAO/WHO regulations and have evaluated whether GMOs and derived foods were substantial equivalents to mother plants or not [5,6]. To date, virtually no harmful effects have been observed in these studies focusing on GM crops, including corn [34], soybeans [35], and rice [36]. Previously, 44 peer reviewed articles describing comparative compositions and 90-day toxicity feeding studies for nine crops since 1995 and 60 opinions of the EFSA have included such tests [37]. None of these studies concluded that there were any safety problems. Although the results of current studies have been published, there are still overwhelming doubts about GM foods [22].

For food safety concerns, the composition of soybeans is also an important factor in safety assessment. A previous report indicated that the nutrient as well as anti-nutrient contents were substantially equivalent to those of non-GM counterparts and within the range of reported values for other commercial soybean lines [38]. Although these results are not sufficient to conclude that feeding GM soybeans has no negative effects on rats, this repeated dose 90-day feeding toxicity trial of GM soybeans shows no adverse effects in rats and that GM soybeans can be recognized as an equivalent and safe substitute for non-GM soybeans. In addition, raw soybeans also contain some anti-nutritional factors, such as trypsin inhibitors, lectins, α-amylase inhibiting factor, goitrin, and soybean antigens, etc. These anti-nutritional factors lead to decreased feed intake, growth and intestinal proteolysis, and increased metabolic disease [39].

Despite the fact that the content of trypsin inhibitor was different in GM and non-GM soybeans, trypsin inhibitor activity was decreased as the temperature increased [40]. In our study, two commercial GM soybeans and one non-GM soybean were used to perform a 90-day feeding toxicity study to elucidate the potential for unintended effects in terms of toxicological and/or nutritional relevance to establish the no observed adverse effect levels (NOAEL) and to determine whether GM soybeans are substantial equivalents to non-GM soybeans. In order to mimic the eating habits in Taiwan, soybean samples were steamed for 20 min at 121 °C by autoclave and dissolved in water. According to the Taiwan food balance sheet in 2020, the daily intake of soybeans is 0.51 g/kg for humans [3] and we selected a low dosage of 1 g/kg and a high dosage of 5 g/kg of soybeans to conduct the food safety in rats. The dosage levels administered were higher than those for human consumption in Taiwan. Although some test data revealed statistical differences in feed consumption, hematology, coagulation, and serum chemistry parameters between non-GM soybean and GM soybean fed groups, no biologically significant differences were observed, and these data were still within the normal range [28]. Furthermore, although some non-specific lesions were randomly found in the non-GM and GM soybean-treated groups, no obvious gross or microscopic lesions were found in male or female rats.

## 5. Conclusions

Our results reveal that no pathological side effects attributable to non-GM, GM-1 or GM-2 soybean fed rats were observed. In this study, the results showed that a 90-day feeding toxicity test did not find any evidence of adverse health effects in male or female rats following 90 consecutive days of gavage with GM soybeans, and concluded that commercial GM soybeans are substantially equivalent to non-GM soybeans.

Although there is no evidence that commercial GM crops and their products have food safety issues, in order to reduce the safety concerns of consumers about this modern biotechnology-derived food, it is still necessary to continuously conduct market monitoring and safety assessment of related products. In this study, commercialized GM soybeans were used for the toxicological assessment in a whole food feeding trial. This not only complies with the regulations of EFSA but also related methods can point out the differences between the global regulatory system and research science.

## Figures and Tables

**Table 1:** Feed consumption and feed efficiency of rats fed with commercial non-GM, GM-1, and GM-2 soybeans for 90 days.

Group/Week	Feed Consumption (g/day) ^a^					
	Non-GM		GM-1		GM-2	
	1 g/kg	5 g/kg	1 g/kg	5 g/kg	1 g/kg	5 g/kg
Male						
1	25.5 ± 2.5	23.7 ± 3.2	25.9 ± 2.1	24.8 ± 2.0	27.1 ± 2.1	25.0 ± 2.0
2	26.1 ± 1.9	24.5 ± 2.8	26.9 ± 2.4	24.1 ± 4.	28.3 ± 1.9 *	24.1 ± 3.7
3	26.3 ± 3.1	25.9 ± 2.9	28.3 ± 2.1	27.5 ± 3.7	29.4 ± 1.9 *	25.3 ± 3.4
4	27.1 ± 2.4	26.0 ± 2.6	29.2 ± 1.6 *	27.1 ± 3.4	30.1 ± 1.7 *	26.2 ± 2.9
5	27.6 ± 2.8	26.1 ± 2.3	30.2 ± 1.0 *	26.6 ± 3.0	30.8 ± 2.6 *	26.9 ± 4.2
6	28.8 ± 2.4	25.3 ± 2.0	31.0 ± 1.3 *	26.6 ± 3.4	31.3 ± 2.3 *	25.9 ± 3.4
7	27.4 ± 3.3	24.0 ± 1.9	27.6 ± 1.0	24.9 ± 3.3	27.8 ± 2.4	24.5 ± 2.8
8	27.7 ± 2.6	24.4 ± 2.2	29.5 ± 1.0	26.3 ± 3.0	29.3 ± 1.5	25.4 ± 2.1
9	28.4 ± 4.7	23.6 ± 2.3	29.5 ± 2.3	25.5 ± 3.5	30.5 ± 3.1	24.3 ± 2.3
10	26.9 ± 3.9	25.4 ± 4.1	29.6 ± 1.3	26.7 ± 4.9	30.9 ± 3.2 *	24.8 ± 2.4
11	27.4 ± 3.9	23.8 ± 2.8	29.1 ± 1.1	24.8 ± 2.8	30.0 ± 2.8	24.2 ± 4.1
12	28.5 ± 3.4	24.5 ± 2.7	26.7 ± 9.5	26.0 ± 2.9	30.1 ± 2.4	23.8 ± 1.7
13	24.3 ± 4.2	20.3 ± 2.3	26.1 ± 2.8	21.9 ± 3.1	27.6 ± 4.3	20.7 ± 2.3
Feed efficiency (%) ^b^	13.9 ± 0.9	14.4 ± 0.8	15.1 ± 1.4 *	14.4 ± 2.0	14.6 ± 0.7	13.2 ± 2.0
Female						
1	20.1 ± 1.4	18.9 ± 1.5	21.8 ± 2.3 *	20.3 ± 2.5	20.7 ± 1.6	20.0 ± 1.8
2	20.0 ± 3.0	17.3 ± 2.4	18.4 ± 4.9	19.4 ± 3.4	18.8 ± 2.0	19.9 ± 2.6
3	20.5 ± 2.0	18.2 ± 2.6	21.0 ± 2.9	19.8 ± 2.8	20.2 ± 2.8	20.0 ± 2.3
4	21.7 ± 2.5	20.5 ± 4.6	21.4 ± 4.3	22.9 ± 4.5	19.7 ± 3.2	19.9 ± 1.8
5	22.2 ± 4.5	19.6 ± 2.3	21.9 ± 3.0	21.3 ± 4.3	19.7 ± 2.0	20.1 ± 2.3
6	26.1 ± 2.0	25.3 ± 2.4	28.9 ± 2.8	26.6 ± 2.6	24.4 ± 4.3	26.8 ± 1.5
7	27.4 ± 3.1	25.1 ± 2.4	26.9 ± 2.1	25.5 ± 1.7	25.9 ± 1.9	25.3 ± 1.9
8	26.0 ± 1.6	24.4 ± 2.5	25.4 ± 2.4	24.7 ± 1.9	24.9 ± 2.1	23.4 ± 2.5
9	27.6 ± 2.4	26.4 ± 2.5	27.3 ± 2.5	26.7 ± 3.0	26.7 ± 2.0	26.7 ± 1.3
10	26.6 ± 2.2	24.2 ± 3.1	27.3 ± 2.3	24.7 ± 2.0	25.6 ± 2.0	24.8 ± 2.5
11	26.6 ± 1.3	25.2 ± 2.0	26.4 ± 1.9	24.5 ± 2.0	24.9 ± 2.1 *	24.9 ± 2.2
12	26.4 ± 2.5	24.6 ± 3.2	26.3 ± 2.2	24.9 ± 2.2	26.6 ± 2.0	25.6 ± 2.7
13	23.9 ± 3.1	22.4 ± 2.2	23.6 ± 1.5	21.4 ± 1.4	22.5 ± 2.1	23.2 ± 1.3
Feed efficiency (%)	6.5 ± 1.4	6.4 ± 0.9	6.8 ± 1.2	6.4 ± 1.0	6.8 ± 1.0	6.8 ± 1.1

Data are expressed as the mean ± SD (n = 9–10). ^a^ Feed consumption (g/day) = [Total feed intake (g)/test period (day)]. ^b^ Feed efficiency (%) = [Daily body weight gain (g)/daily feed intake (g)] × 100. * Significant difference between the same dosage of non-GM and GM groups at *p* < 0.05.

**Table 2:** Hematology of rats fed with commercial non-GM, GM-1, and GM-2 soybeans for 90 days.

Group/Items ^a^	Non-GM (g/kg)		GM-1 (g/kg)		GM-2 (g/kg)	
	1	5	1	5	1	5
Male						
RBC (10^6^/μL)	8.7 ± 0.5	8.5 ± 0.4	8.5 ± 0.6	8.7 ± 0.2	9.0 ± 0.3	9.2 ± 0.3 *
HGB (g/dL)	16.0 ± 0.5	15.2 ± 0.4	15.8 ± 1.0	15.7 ± 0.5 *	16.5 ± 0.7	16.7 ± 0.5 *
HCT (%)	46.4 ± 1.5	44.4 ± 1.2	45.8 ± 2.7	46.2 ± 1.6 *	48.6 ± 2.0 *	48.8 ± 2.7 *
MCV (fL)	53.5 ± 1.9	52.6 ± 2.2	53.7 ± 1.6	53.4 ± 1.2	54.1 ± 2.6	53.0 ± 2.1
MCH (pg)	18.5 ± 0.8	17.9 ± 0.7	18.6 ± 0.9	18.1 ± 0.4	18.4 ± 0.9	18.2 ± 0.5
MCHC (g/dL)	34.5 ± 0.6	34.1 ± 1.0	34.6 ± 1.7	34.0 ± 1.4	34.0 ± 0.9	34.3 ± 1.1
PLT (10^3^/μL)	674 ± 77	651 ± 103	686 ± 166	711 ± 161	636 ± 112	640 ± 90
PT (s)	10.9 ± 0.2	11.5 ± 1.8	10.7 ± 0.5	12.4 ± 3.3	11.6 ± 2.2	11.7 ± 1.1
APTT (s)	23.5 ± 3.9	28.6 ± 11.3	26.3 ± 4.9	31.6 ± 11.2	30.4 ± 6.5 *	26.8 ± 3.5
FBG (mg/dL)	223.9 ± 18.7	187.1 ± 48.0	233.2 ± 34.3	207.6 ± 26.1	219.8 ± 51.2	205.2 ± 57.7
Female						
RBC (10^6^/μL)	8.0 ± 0.2	7.9 ± 0.4	8.3 ± 0.4	8.3 ± 0.4 *	8.0 ± 0.3	8.2 ± 0.4
HGB (g/dL)	15.2 ± 0.5	15.2 ± 0.7	15.5 ± 0.6	15.5 ± 0.6	15.0 ± 0.7	15.4 ± 1.2
HCT (%)	44.8 ± 1.5	45.4 ± 1.7	46.5 ± 2.4	46.7 ± 1.9	44.9 ± 2.3	46.8 ± 2.3
MCV (fL)	56.0 ± 1.0	57.7 ± 2.0	56.2 ± 2.0	56.4 ± 2.4	56.4 ± 2.7	57.1 ± 2.1
MCH (pg)	18.9 ± 0.5	19.3 ± 0.7	18.7 ± 0.5	18.7 ± 0.9	18.8 ± 0.7	18.8 ± 1.6
MCHC (g/dL)	33.8 ± 0.6	33.4 ± 0.8	33.3 ± 0.8	33.1 ± 1.2	33.4 ± 0.7	32.8 ± 2.6
PLT (10^3^/μL)	625.0 ± 105.0	619.1 ± 149.9	650.3 ± 137.8	488.6 ± 169.2	578.7 ± 113.7	556.7 ± 164.5
PT (s)	9.8 ± 0.3	10.3 ± 1.4	10.0 ± 0.5	10.8 ± 1.6	10.0 ± 0.4	10.2 ± 0.3
APTT (s)	21.5 ± 3.1	23.6 ± 5.0	23.4 ± 2.6	30.3 ± 8.6	25.5 ± 6.1	28.6 ± 5.3 *
FBG (mg/dL)	184.2 ± 19.4	154.8 ± 31.3	172.1 ± 27.1	158.0 ± 22.2	177.9 ± 37.9	147.2 ± 35.3

Data are expressed as the mean ± SD (n = 9–10). ^a^ RBC, red blood cell; HGB, hemoglobin; HCT, hematocrit; MCV, mean corpuscular volume; MCH, mean corpuscular hemoglobin; MCHC, mean corpuscular hemoglobin concentration; PLT, platelets; PT, prothrombin time; APTT, activated partial thromboplastin time; FBG, fibrinogen. * Significant difference between the same dosage of non-GM and GM groups at *p* < 0.05.

**Table 3:** White blood cell differentiation of rats fed with commercial non-GM, GM-1, and GM-2 soybeans for 90 days.

Group ^a^/Items	Non-GM (g/kg)		GM-1 (g/kg)		GM-2 (g/kg)	
	1	5	1	5	1	5
Male						
WBC (10^3^/μL)	7.3 ± 1.8	4.5 ± 1.4	5.6 ± 1.3 *	4.7 ± 0.9	5.1 ± 1.6 *	5.9 ± 1.7
Lymphocyte (%)	84.6 ± 9.3	77.4 ± 8.0	79.2 ± 12.0	81.0 ± 10.7	76.9 ± 11.0	84.3 ± 9.7
Neutrophil						
Band (%)	0.0 ± 0.0	0.1 ± 0.3	0.0 ± 0.0	0.0 ± 0.0	0.0 ± 0.0	0.0 ± 0.0
Segment (%)	11.8 ± 7.7	19.6 ± 7.3	17.3 ± 12.7	16.2 ± 10.1	19.8 ± 11.2	12.9 ± 8.7
Monocyte (%)	2.4 ± 1.6	1.7 ± 1.5	2.2 ± 1.4	1.6 ± 1.3	2.8 ± 1.4	2.2 ± 1.6
Eosinophil (%)	1.2 ± 1.1	1.2 ± 0.8	0.9 ± 0.8	1.2 ± 1.5	0.5 ± 0.7	0.4 ± 0.7 *
Basophil (%)	0.0 ± 0.0	0.0 ± 0.0	0.0 ± 0.0	0.0 ± 0.0	0.0 ± 0.0	0.0 ± 0.0
Female						
WBC (10^3^/μL)	4.7 ± 1.8	4.0 ± 1.3	3.9 ± 0.9	3.8 ± 1.5	4.6 ± 1.5	4.1 ± 2.0
Lymphocyte (%)	80.6 ± 9.8	81.0 ± 5.8	80.0 ± 4.3	84.5 ± 10.2	80.5 ± 8.3	75.5 ± 6.5
Neutrophil						
Band (%)	0.0 ± 0.0	0.0 ± 0.0	0.0 ± 0.0	0.1 ± 0.3	0.0 ± 0.0	0.1 ± 0.3
Segment (%)	16.5 ± 9.3	16.9 ± 5.3	16.7 ± 3.8	12.9 ± 9.4	15.7 ± 8.9	20.8 ± 6.0
Monocyte (%)	2.5 ± 1.2	2.0 ± 0.7	2.3 ± 1.3	2.0 ± 1.2	2.9 ± 0.9	3.0 ± 1.2 *
Eosinophil (%)	0.4 ± 0.4	0.1 ± 0.3	1.0 ± 1.2	0.4 ± 0.8	0.3 ± 0.7	0.6 ± 0.7 *
Basophil (%)	0.0 ± 0.0	0.0 ± 0.0	0.0 ± 0.0	0.0 ± 0.0	0.0 ± 0.0	0.0 ± 0.0

Data are expressed as the mean ± SD (n = 9–10). ^a^ WBC, white blood count. * Significant difference between the same dosage of non-GM and GM groups at *p* < 0.05.

**Table 4:** Serum biochemistry of male rats fed with commercial Non-GM, GM-1, and GM-2 soybeans for 90 days.

Group/Items ^a^	Non-GM (g/kg)		GM-1 (g/kg)		GM-2 (g/kg)	
	1	5	1	5	1	5
Male						
Albumin (g/dL)	3.7 ± 0.1	3.5 ± 0.3	3.7 ± 0.1	3.7 ± 0.2 *	3.7 ± 0.1	3.7 ± 0.3
ALP (U/L)	91.0 ± 18.5	99.9 ± 35.9	95.1 ± 22.9	94.0 ± 34.8	81.4 ± 23.0	91.6 ± 23.5
Amylase (U/L)	1965.6 ± 359.2	1455.0 ± 397.1	1903.9 ± 425.2	1648.5 ± 345.9	1818.6 ± 241.7	1589.6 ± 307.9
ALT (U/L)	158.7 ± 40.4	150.6 ± 72.6	140.6 ± 42.2	150.7 ± 41.9	143.3 ± 52.4	164.3 ± 56.4
AST (U/L)	42.0 ± 27.3	41.4 ± 7.5	36.4 ± 7.4	43.1 ± 8.9	34.2 ± 5.6	39.4 ± 9.0
BUN (mg/dL)	18.2 ± 3.9	19.8 ± 4.7	19.8 ± 4.5	18.7 ± 3.5	20.3 ± 5.0	21.0 ± 6.6
Creatinine (mg/dL)	0.3 ± 0.1	0.4 ± 0.2	0.3 ± 0.1	0.4 ± 0.1	0.4 ± 0.1 *	0.4 ± 0.1
CK (U/L)	1046.3 ± 257.8	678.0 ± 273.1	922.8 ± 548.1	795.1 ± 216.2	808.7 ± 482.8	919.7 ± 398.5
Glucose (mg/dL)	127.3 ± 32.4	128.1 ± 34.6	116.9 ± 32.4	143.4 ± 57.3	113.2 ± 38.0	104.1 ± 40.8
LDH (U/L)	2201 ± 707	1235 ± 377	1732 ± 711	1337 ± 346	1729 ± 1034	1991 ± 854 *
TC (mg/dL)	62.0 ± 17.2	51.9 ± 7.5	68.0 ± 14.9	59.4 ± 16.4	70.3 ± 15.6	52.9 ± 13.5
TG (mg/dL)	46.4 ± 29.5	25.2 ± 8.8	32.2 ± 10.1	33.8 ± 12.9	41.1 ± 10.9	37.4 ± 10.2 *
TP (g/dL)	5.8 ± 0.3	5.5 ± 0.5	6.0 ± 0.4	5.8 ± 0.3	5.9 ± 0.2	5.9 ± 0.5
Ca^2+^ (mg/dL)	9.6 ± 0.7	9.5 ± 0.5	9.5 ± 0.6	9.7 ± 0.5	9.9 ± 0.5	9.9 ± 0.3
Cl^−^ (mEq/dL)	103.3 ± 4.4	103.8 ± 3.0	104.1 ± 2.6	103.7 ± 3.2	104.7 ± 2.8	105.2 ± 3.9
K^+^ (mEq/dL)	9.7 ± 1.8	10.0 ± 1.3	9.5 ± 1.6	10.0 ± 1.4	9.8 ± 1.3	9.7 ± 1.2
Mg^2+^ (mEq/dL)	2.3 ± 0.4	2.2 ± 0.3	2.4 ± 0.4	2.3 ± 0.3	2.6 ± 0.4	2.4 ± 0.4
Na^+^ (mEq/dL)	140.6 ± 1.5	140.1 ± 2.2	140.6 ± 1.7	140.9 ± 2.6	141.8 ± 2.7	141.4 ± 3.1
P^3−^ (mg/dL)	7.9 ± 1.8	8.0 ± 1.1	8.6 ± 2.3	8.1 ± 1.4	9.1 ± 1.2	8.8 ± 1.6

Data are expressed as the mean ± SD (n = 9–10). a Tables may have a footer. ALP: alkaline phosphatase; ALT: alanine aminotransferase; AST: aspartate aminotransferase; BUN: blood urea nitrogen; CK: creatine kinase; LDH: lactate dehydrogenase; TC: total cholesterol; TG: triglyceride; TP: total protein. * Significant difference between the same dosage of non-GM and GM groups at *p* < 0.05.

**Table 5:** Serum biochemistry of female rats fed with commercial non-GM, GM-1, and GM-2 soybeans for 90 days.

Group/Items ^a^	Non-GM (g/kg)		GM-1 (g/kg)		GM-2 (g/kg)	
	1	5	1	5	1	5
Female						
Albumin (g/dL)	3.7 ± 0.4	3.5 ± 0.2	3.8 ± 0.2	3.7 ± 0.2 *	3.8 ± 0.3	3.7 ± 0.2 *
ALP (U/L)	53.7 ± 9.8	69.0 ± 26.7	61.2 ± 12.1	49.7 ± 19.6	47.6 ± 11.2	62.0 ± 24.7
Amylase (U/L)	1253.4 ± 285.6	1098.3 ± 123.8	1272.5 ± 315.2	1145.9 ± 162.9	1184.8 ± 246.5	1248.8 ± 262.8
ALT (U/L)	116.3 ± 20.4	99.2 ± 26.4	120.0 ± 15.1	164.6 ± 126.2	120.4 ± 35.0	126.7 ± 32.0
AST (U/L)	44.3 ± 26.2	27.6 ± 6.2	27.9 ± 4.5	30.3 ± 12.4	38.3 ± 23.9	31.3 ± 6.4
BUN (mg/dL)	18.3 ± 4.3	21.2 ± 3.9	18.5 ± 2.6	22.4 ± 4.0	20.2 ± 4.5	20.4 ± 3.4
Creatinine (mg/dL)	0.4 ± 0.1	0.4 ± 0.1	0.4 ± 0.1	0.5 ± 0.1 *	0.4 ± 0.0	0.5 ± 0.1 *
CK (U/L)	479.9 ± 195.4	477.1 ± 261.8	663.7 ± 239.1	1069.2 ± 1451.9	503.4 ± 168.5	648.8 ± 437.6
Glucose (mg/dL)	97.0 ± 22.0	95.1 ± 15.3	81.3 ± 14.1	84.1 ± 15.5	94.5 ± 28.2	77.5 ± 21.6 *
LDH (U/L)	898.9 ± 350.4	929.7 ± 557.5	1174.2 ± 308.9	1357.4 ± 796.3	987.9 ± 356.4	1176.0 ± 730.9
TC (mg/dL)	69.6 ± 9.6	49.5 ± 10.5	61.1 ± 11.1	60.9 ± 10.2	69.7 ± 21.1	71.1 ± 21.0 *
TG (mg/dL)	33.1 ± 9.7	29.9 ± 8.5	29.8 ± 7.6	34.3 ± 8.9	35.5 ± 10.4	32.4 ± 10.4
TP (g/dL)	6.0 ± 0.5	5.7 ± 0.3	6.0 ± 0.4	5.9 ± 0.3	5.9 ± 0.4	5.9 ± 0.3
Ca^2+^ (mg/dL)	9.6 ± 0.6	9.8 ± 0.4	9.7 ± 0.3	9.6 ± 0.3	9.7 ± 0.4	9.7 ± 0.2
Cl^−^ (mEq/dL)	106.2 ± 2.4	106.1 ± 3.7	105.8 ± 3.3	105.6 ± 3.1	105.5 ± 2.6	103.9 ± 2.8
K^+^ (mEq/dL)	8.8 ± 1.7	8.3 ± 1.2	8.6 ± 1.5	8.4 ± 1.4	8.9 ± 1.3	7.7 ± 1.2
Mg^2+^ (mEq/dL)	2.3 ± 0.3	2.3 ± 0.3	2.4 ± 0.3	2.5 ± 0.3	2.7 ± 0.3 *	2.5 ± 0.2
Na^+^ (mEq/dL)	140.2 ± 3.0	139.9 ± 3.0	139.2 ± 3.6	139.7 ± 3.1	139.0 ± 3.1	138.3 ± 2.3
P^3−^ (mg/dL)	7.0 ± 1.4	9.2 ± 2.2	7.6 ± 1.4	9.2 ± 2.2	8.7 ± 1.9 *	8.3 ± 1.4

Data are expressed as the mean ± SD (n = 9–10). ^a^ ALP: alkaline phosphatase; ALT: alanine aminotransferase; AST: aspartate aminotransferase; BUN: blood urea nitrogen; CK: creatine kinase; LDH: lactate dehydrogenase; TC: total cholesterol; TG: triglyceride; TP: total protein. * Significant difference between the same dosage of non-GM and GM groups at *p* < 0.05.

**Table 6:** Relative organ weight (%) of rats fed with commercial non-GM, GM-1, and GM-2 soybeans for 90 days.

Group/Organ ^a^	Non-GM (g/kg)		GM-1 (g/kg)		GM-2 (g/kg)	
	1	5	1	5	1	5
Male						
Adrenal (%)	0.08 ± 0.02	0.08 ± 0.01	0.08 ± 0.01	0.10 ± 0.02 *	0.08 ± 0.02	0.11 ± 0.02 *
Brain (%)	0.4 ± 0.0	0.4 ± 0.0	0.4 ± 0.0	0.4 ± 0.0	0.4 ± 0.0	0.4 ± 0.0
Heart (%)	0.3 ± 0.0	0.3 ± 0.0	0.3 ± 0.0	0.3 ± 0.0	0.3 ± 0.0	0.3 ± 0.0
Liver (%)	2.4 ± 0.3	2.2 ± 0.2	2.4 ± 0.1	2.4 ± 0.2	2.3 ± 0.2	2.2 ± 0.2
Kidney (%)	0.6 ± 0.0	0.7 ± 0.1	0.6 ± 0.0	0.6 ± 0.1	0.6 ± 0.0	0.6 ± 0.1
Spleen (%)	0.1 ± 0.0	0.1 ± 0.0	0.1 ± 0.0	0.1 ± 0.0	0.1 ± 0.0	0.1 ± 0.0
Thymus (%)	0.1 ± 0.0	0.1 ± 0.0	0.1 ± 0.0	0.1 ± 0.0	0.1 ± 0.0	0.1 ± 0.0
Testes (%)	0.6 ± 0.0	0.7 ± 0.1	0.6 ± 0.0	0.7 ± 0.1	0.6 ± 0.1	0.7 ± 0.1
Female						
Adrenal (%)	0.18 ± 0.01	0.2 ± 0.05	0.18 ± 0.06	0.18 ± 0.06	0.20 ± 0.05	0.19 ± 0.05
Brain (%)	0.7 ± 0.1	0.7 ± 0.1	0.7 ± 0.1	0.7 ± 0.1	0.7 ± 0.1	0.6 ± 0.1
Heart (%)	0.3 ± 0.0	0.3 ± 0.1	0.3 ± 0.1	0.3 ± 0.1	0.3 ± 0.0	0.3 ± 0.0
Liver (%)	2.4 ± 0.3	2.3 ± 0.4	2.3 ± 0.4	2.4 ± 0.5	2.4 ± 0.3	2.2 ± 0.4
Kidney (%)	0.6 ± 0.0	0.7 ± 0.1	0.6 ± 0.1	0.6 ± 0.1	0.7 ± 0.1	0.6 ± 0.1
Spleen (%)	0.2 ± 0.0	0.1 ± 0.0	0.2 ± 0.0	0.2 ± 0.0	0.2 ± 0.0	0.2 ± 0.1
Thymus (%)	0.1 ± 0.0	0.1 ± 0.0	0.1 ± 0.0	0.1 ± 0.0	0.1 ± 0.0	0.1 ± 0.0
Ovary (%)	0.02 ± 0.0	0.03 ± 0.0	0.02 ± 0.0	0.02 ± 0.0	0.03 ± 0.0	0.02 ± 0.0

Data are expressed as the mean ± SD (n = 9–10). ^a^ Relative organ weight (%) = (organ weight (g)/final body weight (g)) × 100. * Significant difference between the same dosage of non-GM and GM groups at *p* < 0.05.

**Table 7:** Summary of pathological changes in rats fed with commercial non-GM, GM-1, and GM-2 soybeans for 90 days.

Organ	Histopathological Findings	Group (5 g/kg)					
		Male			Female		
		Non- GM	GM -1	GM -2	Non -GM	GM -1	GM -2
Adrenal gland		-	-	-	-	-	-
Aorta		-	-	-	-	-	-
Brain							
Fore		-	-	-	-	-	-
Middle		-	-	-	-	-	-
Cerebellum		-	-	-	-	-	-
Stem		-	-	-	-	-	-
Bone, femur		-	-	-	-	-	-
Bone marrow							
Femur and sternum		-	-	-	-	-	-
Cervix		N	N	N	-	-	-
Epididymis		-	-	-	-	-	-
Esophagus		-	-	-	-	-	-
Eyes		-	-	-	-	-	-
Harderian gland							
	Infiltration, mononuclear cell, focal, minimal to slight ^a^	-	-	1/9 ^b^	1/10	-	-
Heart							
	Infiltration, mononuclear cell, focal, minimal to slight	-	-	1/9	-	-	-
Intestine, small							
Duodenum		-	-	-	-	-	-
Jejunum		-	-	-	-	-	-
Ileum		-	-	-	-	-	-
Intestine, large							
Caecum		-	-	-	-	-	-
Colon		-	-	-	-	-	-
Rectum		-	-	-	-	-	-
Kidney							
	Cyst, tubule, focal, slight to moderate	2/9	-	-	-	-	-
Liver		-	-	-	-	-	-
Lung							
	Granuloma, foreign body, focal, slight	1/9	-	-	-	-	-
Lymph node							
Cervical		-	-	-	-	-	-
Mesenteric		-	-	-	-	-	-
Mammary gland		N	N	N	-	-	-
Optic nerve		-	-	-	-	-	-
Ovary		N	N	N	-	-	-
Oviduct		N	N	N	-	-	-
Pancreas							
	Fibrosis, islet, focal, slight	-	-	1/9	-	-	-
Pituitary		-	-	-	-	-	-
Parathyroid gland		-	-	-	-	-	-
Prostate gland					N	N	N
	Infiltration, mononuclear cell, focal, minimal to moderate	4/9	1/10	2/9			
Salivary gland							
Mandibular lobe		-	-	-	-	-	-
Sublingual lobe		-	-	-	-	-	-
Sciatic nerve		-	-	-	-	-	-
Seminal vesicle		-	-	-	N	N	N
Skeletal muscle		-	-	-	-	-	-
Skin		-	-	-	-	-	-
Spinal cord							
Cervical		-	-	-	-	-	-
Lumbar		-	-	-	-	-	-
Thoracic		-	-	-	-	-	-
Spleen							
	Fibrosis, serosa, focal, minimal	-	1/10	-	-	1/10	-
Stomach		-	-	-	-	-	-
Testes		-	-	-	-	-	-
Thymus		-	-	-	-	-	-
Thyroid gland		-	-	-	-	-	-
Tongue		-	-	-	-	-	-
Trachea		-	-	-	-	-	-
Urinary bladder		-	-	-	-	-	-
Uterus		N	N	N	-	-	-
Vagina		N	N	N	-	-	-

- No effect. N: no tissue available. ^a^ Degree of lesions was graded from one to five depending on severity: 1 = minimal (<1%); 2 = slight (1–25%); 3 = moderate (26–50%); 4 = moderate/severe (51–75%); 5 = severe/high (76–100%). ^b^ Incidence: Affected rats/Total examined rats (n = 9–10).

## Abbreviations

GM, genetically modified; GMO, genetically modified organism; PCR, polymerase chain reaction; RBC, red blood cell; HGB, hemoglobin; HCT, hematocrit; MCV, mean corpuscular volume; MCH, mean corpuscular hemoglobin; MCHC, mean corpuscular hemoglobin concentration; PLT, platelet; FBG, fibrinogen; WBC, white blood cell; ALP, alkaline phosphatase; ALT, alanine aminotransferase; AST, aspartate aminotransferase; BUN, blood urea nitrogen; CK, creatine kinase; LDH, lactate dehydrogenase, TC, total cholesterol; TG, triglycerides; TP, total protein; EPSPS, 5-enolpyruvoylshikimate-3-phosphate synthase; PAT, phosphinothricin acetyltransferase; SD, standard deviation.
